# Serum endocan as a predictive biomarker of cardiovascular risk in obese pediatric patients

**DOI:** 10.1186/s13052-023-01510-y

**Published:** 2023-08-31

**Authors:** Carmela Morace, Selenia Lorenza Curatola, Giuseppe Mandraffino, Michele Scuruchi, Angela Elvira Serrano’, Angelo Tropeano, Fortunato Lombardo, Giuseppina Salzano, Giovanni Squadrito, Antonio Giovanni Versace, Mariella Valenzise

**Affiliations:** 1 Department of Clinical and Experimental Medicine, University of Messina, Messina, Italy; 2 Department of Human Pathology of Adulthood and Childhood, Unit of Pediatrics, University of Messina, Via Consolare Valeria 98126, Messina, Italy

**Keywords:** Endocan, Obesity, Cardiovascular risk, Children

## Abstract

**Background:**

Endocan is a soluble dermatan sulfate proteoglycan (50 kDa) secreted by endothelial cells and expressed by dermal, coronary, pulmonary and adipose tissue microvasculature. It plays an important role in the pathogenesis of vascular disorders, inflammatory state, endothelium dysfunction and neoangiogenesis. Aims of the study were to compare fasting serum endocan levels between children with obesity and healthy controls and to investigate the relationships between endocan, body mass index (BMI) and other indices of cardiometabolic risk.

**Methods:**

This single-center, observational, retrospective study included 19 pediatric patients with obesity aged 11.94 ± 0.52 years and 19 lean matched controls. Each patient underwent clinical and auxological examination and laboratory investigations including routine organs function tests and lipid profile. Homeostasis model assessment of insulin resistance (HOMA-IR) was calculated. Fasting endocan serum levels were measured using an enzyme-linked immunosorbent assay (ELISA).

**Results:**

Compared to healthy subjects, serum endocan levels were found to be significantly upraised in children with obesity. Endocan resulted significantly correlated with insulin levels (rho 0.47; *p* = 0.04); in addition, an association with HOMA-IR values with a trend toward the statistical significance (rho 0.43; *p* = 0.07) was found. No significant correlation with fasting blood glucose values and lipid serum levels was demonstrated. Although not statistically significant, a correlation between endocan and the presence and grading of liver steatosis on ultrasound (rho 0.51; *p* = 0.08 and rho 0.51; *p* = 0.08, respectively) was found.

**Conclusions:**

These findings confirm the association between endothelial damage and insulin resistance in children with obesity. Endocan could be used as a biomarker of early endothelial dysfunction in children with obesity and could be a valid predictor of future cardiovascular risk in adulthood.

## Background

Endocan, formerly called endothelial cell-specific molecule-1 (ESM-1), is a soluble dermatan sulfate proteoglycan (50 kDa) expressed by endothelial cells of dermal, coronary, pulmonary and adipose tissue microvasculature. It is secreted into the bloodstream, where it is found at concentrations of 0.5–1 ng/ml in the plasma of healthy subjects [1, 2].

Endocan may be involved in molecular interactions with a variety of biologically active modalities essential for the regulation of biological processes related to cell adhesion, migration, proliferation, leukocyte migration, and neovascularization [3]. Endocan also plays a role in the development of vascular disease, inflammation process, and endothelial dysfunction [4]. Moreover it acts on endothelial cells, promoting the action of vascular endothelial growth factor-A (VEGF-A) on its receptor (VEGFR-2) and inducing endothelial permeability [2]. Endocan level is significantly increased in various diseases such as cancer, sepsis, pneumonia, pulmonary thromboembolism, diabetes mellitus, rheumatoid arthritis, osteoarthritis and cardiovascular diseases [3, 4]. In addition, Endocan may have prognostic role for these diseases and patient outcomes [4, 5].

Endocan has been proposed in several studies as a novel endothelial mediator that stimulates the proliferation and migration of vascular smooth muscle cells, and may contribute to neointima formation during atherogenesis, leading to the thickening of the intima-media layer [6]. Thus, endocan with its inflammatory and pro-angiogenic effects may play a role in the process of atherosclerosis [7]. Furthermore endocan levels may reflect a condition of vascular inflammation [8], with possible implications in the pathophysiology of hypertension, as demonstrated in a cohort of adult patients [9]. It is well known that children with obesity are at increased risk of developing hypertension, dyslipidemia, impaired glucose tolerance, insulin resistance or type2 diabetes mellitus and early atherosclerosis, with a consequent increased risk for cardiovascular disease over time [9, 10]. In clinical practice, it would be desirable to stratify this risk with a noninvasive and easy tool in order to obtain prognostic marker and endocan would own all these features.

The aims of present study were 1) to compare fasting serum endocan levels between children with obesity and healthy controls and 2) to investigate the relationships of endocan with body mass index (BMI) and other indices of cardiometabolic risk.

## Methods

### Study design and population

This is a single-center, observational, retrospective study carried out at the Pediatric Endocrinology Outpatient Clinic at the University Hospital of Messina, Italy. Nineteen Caucasian children and adolescents with obesity (9 males and 10 females) were enrolled to the study as admitted to our outpatient clinic for obesity. Inclusion criteria were: BMI ≥  + 2 standard deviation score (SDS) for children with obesity, in accordance with definition of obesity by World Health Organization (WHO) for children from the age of 5 years age ranged between 5 and 16 years [11]; Caucasian ethnicity; BMI SDS included in the normal range according to WHO criteria (BMI SDS > -2 SDS and <  + 2 SDS) was considered for the recruitment of control group patients. Exclusion criteria were: chronic pharmacological therapies; genetic and/or endocrine causes of obesity; family history of dyslipidemia; current or recent inflammatory/infectious diseases.

The control group consisted of patients of the same age range as the study group, female and male with a normal BMI, followed at our Endocrinology Outpatient Clinic for short stature or precocious puberty.

All procedures were performed in accordance with the Declaration of Helsinki and were approved by Ethics Committee of Messina. Written informed consent was obtained from all parents or legal tutors.

### Clinical evaluation

Clinical evaluation was performed according to the following standard procedures: (1) Body weight was determined to the nearest 0.1 kg on accurate and properly calibrated standard beam scales, in minimal underclothes and no shoes; (2) Height was measured to the nearest 0.1 cm on standardized, wall-mounted height boards, according to standardized procedures. The children stood with the head aligned in the Frankfort plane, barefoot, with feet placed together and flat on the ground, heels, buttocks, and scapulae against the vertical backboard, arms loose and relaxed with the palms facing medially; (3) BMI was calculated using the equation: body weight (kg)/height (m)^2^. BMI-SDS values were obtained from the WHO growth references (4; [11]). Waist circumference (WC) was measured at the approximate midpoint between the lower margin of the last palpable rib and the top of the iliac crest; (5) WC-to-height ratio (WHtR) was calculated by dividing WC by height. (6) Diastolic and systolic blood pressure (DBP and SBP) measurements were performed using a mercury sphygmomanometer and the measurements were repeated twice in a sitting position after 30 min of rest, using a cuff appropriate for body size and the average measurement was recorded. The patients underwent a pubertal evaluation assessed by Tanner stages 5 of breast development in girls and testicular volume in boys [12].

### Laboratory and instrumental assessment

A fasting blood sampling for plasma triglycerides, high-density lipoproteins (HDL), low-density lipoproteins (LDL), total cholesterol, glucose, and insulin was performed after overnight fasting (at least 8 h). These parameters were analyzed with standard techniques: triglycerides were measured enzymatically, the HDL-cholesterol fraction was obtained after precipitation using a phosphotungstic reagent, glucose was measured using a glucose oxidase method; serum insulin was determined by a chemiluminescence immunoassay. Liver, and kidney function tests were also performed according to the standard techniques. C-reactive protein (CRP) was measured with an immunoturbidimetric method.

Fasting endocan serum levels were measured using an enzyme-linked immunosorbent assay (ELISA) kit accordingly to the manufacturer’s instructions (the blood samples were centrifuged at 3200 rpm for 10 min at + 4 = C. Subsequently, the serum samples were stored at -80 °C until analysis).

Insulin resistance (IR) was measured through homeostasis model assessment of insulin resistance (HOMA-IR). This index was calculated using the equation: fasting insulin (μU/ml) × fasting glucose (mg/dl)/405 [13].

In order to evaluate hepatosteatosis, children with obesity underwent abdominal ultrasonography, performed by the same radiologist. Steatosis was graded as follows: Absent (grade 0) when the echotexture of the liver is normal; mild (grade 1), when there is a slight and diffuse increase of liver echogenicity with normal visualization of the diaphragm and of the portal vein wall; moderate (grade 2), in case of a moderate increase of liver echogenicity with slightly impaired appearance of the portal vein wall and the diaphragm; severe (grade 3), in case of marked increase of liver echogenicity with poor or no visualization of portal vein wall, diaphragm, and posterior part of the right liver lobe [14].

### Statistical analysis

Variables were summarized as mean ± SD. Groups were compared using the Student t-test. Correlations between variables were assessed using the Pearson test. Linear stepwise multivariate regression analysis was performed to assess the contribution of each variable to the study variables. A two-tailed alpha of 0.05 was used to value statistical significance. IBM SPSS statistics for Mac (ver. 26, IBM Corporation) was utilized to study the statistical data.

## Results

Thirty-eight children were consecutively recruited, including 19 Caucasian children with obesity and 19 normal-weight controls, matched for age (11.94 ± 0.52 vs 10.32 ± 0.96 years; *p* = 0.07), sex (9 males/10 females vs 9 males/10 females). Liver and kidney function tests were normal in the entire population. Table 1 shows the physical and biochemical features of the total sample.

**Table 1:** Physical and biochemical features of the total sample and comparison analysis

	**Cases** ***N* = 19**	**Control group** ***N* = 19**	***p***
AGE	11.94 ± 0.52	9.32 ± 0.96	0.041
BMI	30.04 ± 0.98	15.96 ± 0.40	0.000
WTHR	0.66 ± 0.80	0.38 ± 1.20	0.000
SBP	121.87 ± 2.42	104.50 ± 4.50	0.070
DBP	67.55 ± 1.84	65.11 ± 2.70	0.821
TC mg/dl	175.52 ± 6.37	157.50 ± 11.56	0.194
TG mg/dl	104.45 ± 5.95	79.40 ± 13.70	0.093
HDL mg/dl	47.26 ± 2.26	57.70 ± 6.80	0.015
LDL mg/dl	100.50 ± 7.27	75.46 ± 5.40	0.000
N-HDL mg/dl	120.48 ± 8.07	76.80 ± 6.70	0.000
GLUCOSE mg/dl	82.28 ± 1.71	86.86 ± 2.38	0.143
INSULIN μU/ml	20.97 ± 1.73	9.90 ± 1.44	0.023
HOMA	4.39 ± 0.42	2.16 ± 0.42	0.045
AST U/L	25.27 ± 2.48	24.00 ± 2.70	0.586
ALT U/L	28.24 ± 4.63	14.13 ± 1.91	0.048
AST/ALT U/L	1.17 ± 0.11	1.75 ± 0.11	0.003
GGT U/L	17.53 ± 2.18	13.00 ± 2.08	0.366
ENDOCAN ng/ml	2.03 ± 0.15	1.51 ± 0.12	0.022

*Abbreviations*: *BMI* Body mass index, *SBP* Systolic blood pressure, *DBP* Diastolic blood pressure, *TC* Total cholesterol, *TG* Triglycerides, *HDL* High-density lipoproteins, *LDL* Low-density lipoproteins, *AST* Aspartate aminotransferase, *ALT* Alanine aminotransferase

Children with obesity had higher LDL, n-HDL, insulin, HOMA and alanine aminotransferase (ALT) values than healthy controls; conversely, HDL levels were lower in children with obesity (Table 1).

Fasting endocan concentration was significantly higher in children with obesity compared to controls (2.03 ± 0.15 vs 1.51 ± 0.12 ng/ml; *p* = 0.02) (Table 1).

**Fig. 1:**
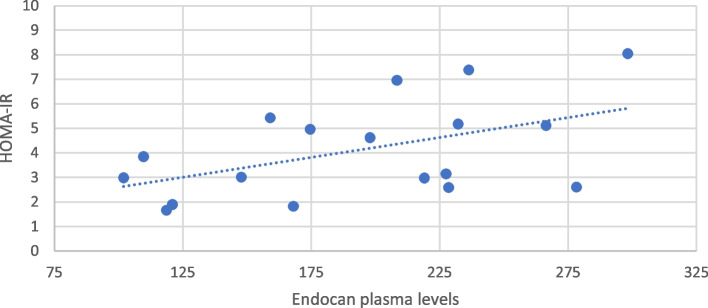
Correlation between endocan and HOMA-IR (Spearman’s Test)

Correlation analysis revealed a positively association only with insulin level (rho = 0.47; *p* = 0.04). No significant correlation with BMI values, fasting glucose and lipid profile was found. However, our data showed the association, with a trend toward the statistical significance, between endocan and HOMA-IR (rho 0.43; *p *= 0.07) ( Fig. 1).

**Fig. 2:**
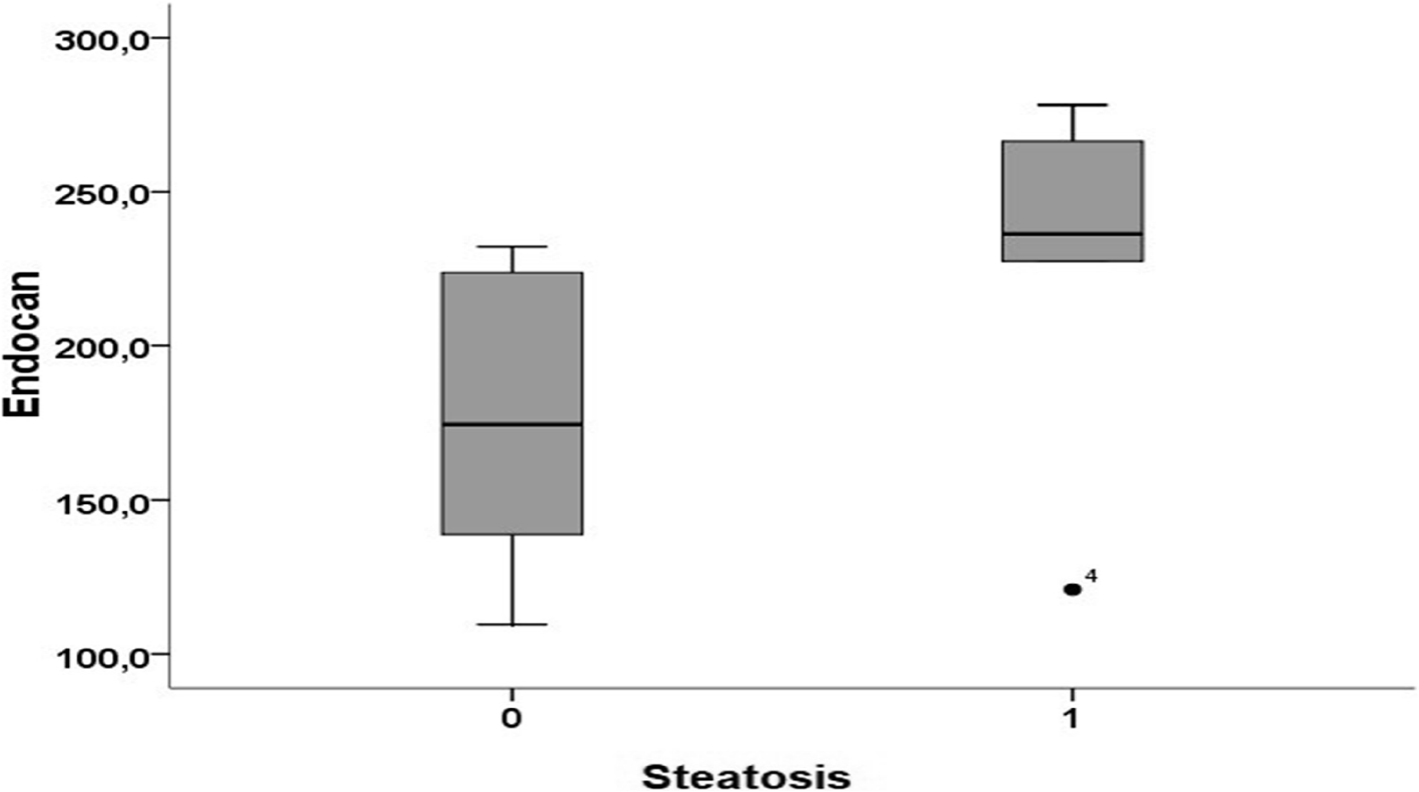
Median levels of Endocan in relation with liver steatosis

Only 12 children with obesity underwent abdomen ultrasound (informed consent was not obtained in 7 cases). The presence of steatosis was reported in 5 cases. No significant correlation between presence and grade of steatosis and endocan levels was found (Table 2 and Fig. 2).

**Table 2:** Correlation panel (Spearman’s test) between endocan and other variables

	**Endocan**	
	Rho	*P*
**AGE**	0.16	0.50
**BMI**	0.35	0.13
**SBP**	-0.11	0.68
**DBP**	-0.27	0.31
**TC** **mg/dl**	0.12	0.67
**TG** **mg/dl**	-0.27	0.34
**HDL** **mg/dl**	-0.11	0.68
**LDL-C** **mg/dl**	0.30	0.21
**NHDL** **mg/dl**	0.20	0.39
**GLUCOSE** **mg/dl**	0.02	0.92
**INSULIN** **μU/ml**	0.47	0.04
***HOMA-IR***	*0.43*	0.07
**STEATOSIS (YES)**	*0.51*	*0.08*
**STEATOSIS GRADING**	*0.51*	*0.08*

*Abbreviations*: *BMI* Body mass index, *SBP* Systolic blood pressure, *DBP* Diastolic blood pressure, *TC* Total cholesterol, *TG* Triglycerides, *NHDL* Non-high-density lipoprotein cholesterol, *LDL* Low-density lipoproteins, *HOMA-IR* Homeostatic Model Assessment for Insulin Resistance

## Discussion

Childhood obesity is one of the major public health problems worldwide rising due to sedentary lifestyles and unhealthy diets. It is important to emphasize that a child with obesity is very likely to become an adult with obesity: the rate of probability increases with age and is directly proportional to the degree of obesity [15]. For a preschooler child with obesity, the probability of becoming an adult with obesity ranges from 26 to 41%; for school-aged children, this percentage reaches 69% and for adolescents with obesity 83% [16]. The association between obesity and increased risk of serious long-term adult complications such as arterial hypertension, dyslipidemia, impaired glucose tolerance, IR, pre-diabetes or type 2 diabetes mellitus and early atherosclerosis is well established [15]. IR is an important link between obesity and associated cardiovascular risk and it has been suggested as one of the early mechanisms for the development of endothelial dysfunction in young individuals with obesity [17, 18]. Indeed, IR is associated with specific central body fat distribution in adolescents with obesity and the onset of metabolic syndrome in childhood [18, 19]. Moreover, recent studies have shown that HOMA-IR levels are closely related to the lipid profile of the pediatric population and that the initial changes in glucose metabolism, found in prepubertal children with obesity, may be one of the factors leading to increased arterial intima thickness [17, 18]. In addition, some studies have shown that adolescents with obesity, with higher levels of intrahepatic steatosis (despite comparable levels of obesity in their peers), have increased fasting glucose levels and systemic IR, and most have significant cardiovascular risk [14, 20, 21].

Furthermore, obesity leads to low-grade chronic inflammation and in obese individuals, in addition to the synthesis of pro-inflammatory cytokines (such as IL-6 and TNFα), a reduction of anti-inflammatory cytokines (such as adiponectin) is observed in adipose tissue [3, 4, 22]. These conditions lead to chronic inflammation and endothelial dysfunction, which form the basis of the atherosclerotic process and increase cardiovascular risk factors [23, 24].

Thus, the close correlation between childhood obesity and cardiovascular risk is well established. For this reason is very important to find an easily accessible marker to stratify cardiovascular risk also for children with obesity. Several studies, as already mentioned, in the medical literature have shown that Endocan plays an important role in the development of vascular disease, inflammation process, and endothelial dysfunction [3, 4, 22]. In our research, in agreement with previous ones, we demonstrated that Endocan serum levels were higher in subjects with obesity compared to the healthy group, moreover, we also found that plasma Endocan levels were significantly related to insulinemia levels. Our results also showed the association, with a trend toward the statistical significance, between endocan and HOMA-IR. We also looked at the relationship between Endocan levels and hepatic steatosis; unfortunately, the small sample size was the main limitation of our research, and not all patients underwent abdominal ultrasound scans, which affected the statistical significance of the data obtained. To date, only another study has examined the association between Endocan and hepatic steatosis in a population of children with obesity, and the results are similar to ours [7]. A previous study in adult patients with obesity, with or without steatosis, reported reduced plasma levels of Endocan compared with healthy lean subjects [10]. The different results between our study and this study may be due to a possible fluctuation in the expression of Endocan during physiological development in children [7]. To date, few studies have been conducted in pediatric populations. This study may provide a basis for future studies.

## Conclusion

Our study demonstrates that Endocan levels are significantly increased in subjects with obesity and that can be correlated with the major metabolic disorders associated with obesity. Although preliminary, our data suggested that Endocan has an important diagnostic and prognostic role that could be used to improve the future management of children with obesity at increased cardio-metabolic risk.

## Data Availability

The datasets used and/or analysed during the current study are available from the corresponding author on reasonable request.

## Abbreviations

GAGs: Glycosaminoglycans
TNF: Tumor necrosis-α
IL1: Interleukin 1
TGF-β1: Transforming growth factor-β1
VEGF: Vascular endothelial growth factor
EGF: Epidermal growth factor
FGF2: Fibroblast growth factor-2
IR: Insulin resistance
